# Influence of Gamma-Phase Aluminum Oxide Nanopowder and Polyester–Glass Recyclate Filler on the Destruction Process of Composite Materials Reinforced by Glass Fiber

**DOI:** 10.3390/polym16162276

**Published:** 2024-08-10

**Authors:** Katarzyna Panasiuk, Krzysztof Dudzik, Grzegorz Hajdukiewicz, Norbert Abramczyk

**Affiliations:** Faculty of Marine Engineering, Gdynia Maritime University, 81-225 Gdynia, Poland; k.dudzik@wm.umg.edu.pl (K.D.); g.hajdukiewicz@wm.umg.edu.pl (G.H.); n.abramczyk@wm.umg.edu.pl (N.A.)

**Keywords:** recycling, composite materials, nanoadditives, tensile strength, acoustic emission, K-S metric entropy

## Abstract

Recycling of composite materials is an important global issue due to the wide use of these materials in many industries. Waste management options are being explored. Mechanical recycling is one of the methods that allows obtaining polyester–glass recyclate in powder form as a result of appropriate crushing and grinding of waste. Due to the fact that the properties of composites can be easily modified by adding various types of fillers and nanofillers, this is one of the ways to improve the properties of such complex composite materials. This article presents the strength parameters of composites with the addition of fillers in the form of polyester–glass recyclate and a nanofiller in the form of gamma-phase aluminum nanopowder. To analyze the obtained results, Kolmogorov-Sinai (K-S) metric entropy was used to determine the transition from the elastic to the viscoelastic state in materials without and with the addition of nanoaluminum, during a static tensile test. The tests included samples with the addition of fillers and nanofillers, as well as a base sample without any additives. The article presents the strength parameters obtained from a testing machine during a static tensile test. Additionally, the acoustic emission method was used during the research. Thanks to which, graphs of the effective value of the electrical signal (RMS) were prepared as a function of time, the parameters were previously identified as extremely useful for analyzing the destruction process of composite materials. The values obtained from the K-S metric entropy method and the acoustic emission method were plotted on sample stretching graphs. The influence of the nanofiller and filler on these parameters was also analyzed. The presented results showed that the aluminum nanoadditive did not increase the strength parameters of the composite with recyclate as a result of the addition of aluminum nanofiller; however, its addition influenced the operational parameters, which is reflected in a 5% increase in the UTS value (from 55% to 60%).

## 1. Introduction

With the increasing use of glass fiber-reinforced polymer composites (GFRP), the demand for recycling post-exploitation and post-production waste is growing. The use of different types of resins and reinforcement does not facilitate the process of their subsequent recovery. A circular economy introduced in a selected industry, which can help eliminate waste and reuse resources, justifies the use of efficient recycling processes of used GFRP components and production waste. Demand for composite materials in the marine, aerospace, construction, wind power, and automotive industries is expected to continue to grow at a compound annual growth rate (CAGR) of approximately 6%, with the total market growing from USD 26 billion in 2018 to USD 41.4 billion in 2025 [1,2,3]. A particularly serious challenge for the environment is the increasing popularity of GFRP in industrial sectors, in the case of waste, such as waste generated during the production of composites (as much as 40%) and GFRP products decommissioned and placed in landfills. By 2025, the amount of GFRP waste in the world is expected to increase to 20,000. tons per year [4]. One of the very important challenges for the polymer composites market is the recovery and reuse of glass fibers from production waste and end-of-life composites in an environmentally friendly and cost-effective manner. There are many recycling processes and they are still being developed, but there is still no solution that the industry would be convinced of. Available recycling options allow for the recovery and reuse of waste, but the resulting material is not attractive to producers. The use of recyclate in the production process causes a huge decrease in the strength of the glass composite compared to its original form [5]. A breakthrough in the field of recycling can completely change the economics of using such composites; however, it is necessary to develop new technologies and research on these materials to obtain the best possible strength parameters [6,7,8,9].

The composite material is easily modified in terms of strength parameters; research on the impact of individual additives and nanoadditives is being conducted by scientists to determine the impact of additives on selected parameters. One such possibility is to combine laminates with additives in the form of nanofillers to create new materials called nanocomposites. Nanocomposites are materials in which at least one component has dimensions on the nanometer scale, which is called a nanofiller [10,11,12].

Nanofillers can be classified in terms of their chemical nature and type of physical structure, but most often they are classified in terms of the shape of the particles [12,13,14,15]. Nanocomposites are attractive due to the interaction and shaping of the polymer matrix with nanofillers at the molecular level. A small amount of nanofiller, with dimensions below 100 nm, added to the matrix (usually a few percent) can change the selected properties of the composite material. This initiated research with aluminosilicates and substances with a lamellar structure, using many polymers as polymer matrices [16,17,18,19,20,21]. In one study [22], metal-based nanoadditives were used and combined with a PLA matrix, thus examining their influence on the surface properties of the antibacterial activity and mechanical properties of the PLA nanoadditive film. An important aspect in these studies was primarily to determine how the addition of nanoparticles to PLA during the extrusion process affects the chemical composition, morphology and wettability of the surface, and its further impact on the antibacterial effectiveness and mechanical properties of PLA-NPs. Another article [23] presents the influence of multi-wall carbon nanotubes on the mechanical and electrical properties of epoxy resins and epoxy composites. Thanks to the use of carbon nanotubes as polymer reinforcement, higher values of tensile strength and a higher percentage of deformation of the tested materials were achieved [24]. In yet another study [25], the influence of the weight percentage of aluminum oxide nanoparticles (0.25–0.5–0.75–1 wt.) and the particle size of 60 nm mixed with epoxy resin was analyzed. Bending, hardness, tensile, erosion and TGA (thermogravimetry) tests were carried out. Experimental results show an improvement in mechanical behavior using 0.25 wt% particles for both flexural strength by 7% and wear resistance by 67% compared to neat epoxy.

Due to the fact that the properties of composites can be easily modified by adding various types of fillers and nanofillers [26,27,28,29,30], this is one of the ways to improve the properties of such complex composite materials. This article presents the influence of additive fillers in the form of polyester–glass recyclate and nanofillers in the form of gamma-phase aluminum nanopowder on the strength parameters during static tensile tests. To analyze the obtained results, Kolmogorov-Sinai (K-S) metric entropy was used to determine the transition from the elastic to viscoelastic state in materials without and with the addition of nanoaluminum. Additionally, the acoustic emission method was used during the research.

## 2. Materials and Methods

### 2.1. Materials

Polyester-glass laminates with the addition of a filler in the form of gamma-phase aluminum nanopowder (20 µm) produced by POL-AURA (Morąg, Poland) and polyester–glass recyclate were used for the tests. Polyester-glass recyclate was obtained by preliminary grinding of materials using a crusher and then grinding the resulting waste to granulation ≤ 1.2 mm. The size of the recyclate was confirmed using a sieve shaker. The content of reinforcement in the obtained recyclate was verified using the calcining method and was 30%. To reduce the risk of improper saturation of subsequent layers of reinforcement with additives, the manual lamination method was used for production instead of technologies such as vacuum infusion, and the granulation of the recyclate for this technology was too high. Polimal 1094-AWTP polyester resin produced by Organika-Sarzyna S.A. (Nowa Sarzyna, Poland) and glass mats with 450 g/m^2^ density distributed by Laminopol Sp.z.o.o. (Redzikowo, Poland) were used. The matrix additives, such as recycled filler and nano-filler, were combined by physical mixing using an electric mixer. Three research materials were made: A0R0—without the addition of nanofiller and filler, A0R10—with 10% non-filler content in the form of polyester–glass recyclate, and A2R10—with 2% nanopowder content and 10% polyester–glass recyclate content. Table 1 shows the % content (by weight) of combined ingredients.

### 2.2. Sample Preparation

**Figure 1:**
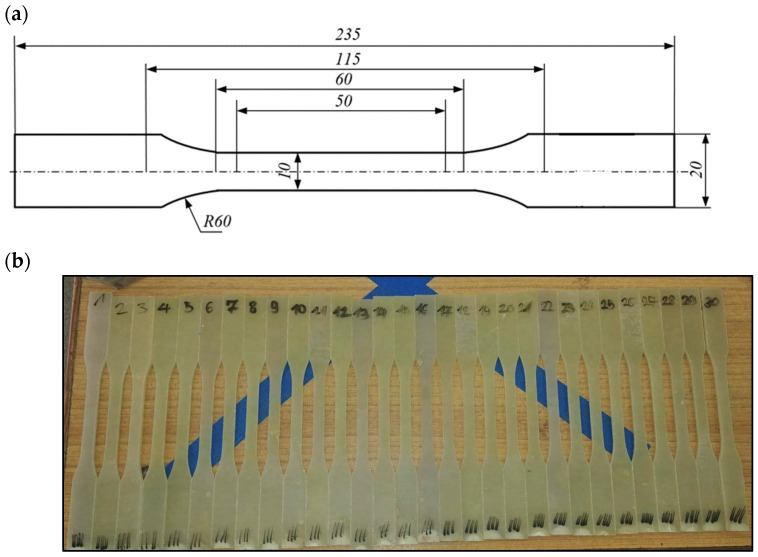
Samples prepared for static tensile test: (**a**) geometric parameters, (**b**) ready for static tensile test.

The composite materials created in the manufacturing process were prepared for static tensile testing in accordance with the PN-EN ISO 527-4 standard [31]. To obtain the appropriate shape of the samples and minimize the influence of cutting temperature, the water-cutting method was used. A total of 30 samples were cut from each material. The average thickness of the samples is: A0R0—5.38 mm, A0R10—6.39 mm, and A2R10 8.45 mm. Figure 1 shows test samples without the addition of fillers.

### 2.3. Methods

**Figure 2:**
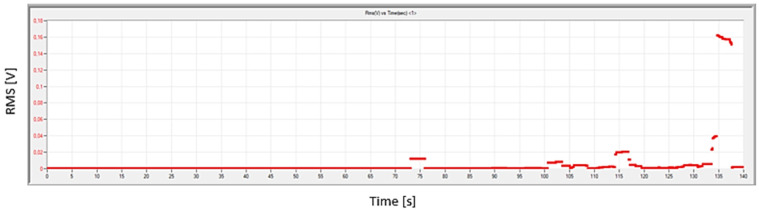
Example sample screenshot from the AE data software.

The use of additional equipment in strength tests allows the determination of many parameters directly related to the qualitative changes occurring in these materials. The acoustic emission method is becoming increasingly widely used in strength tests of many materials and structures [31,32,33]. The analysis of the available literature shows that acoustic emission allows damage detection in materials and is sensitive enough to determine parameters that are not detectable in standard strength tests [34,35,36]. Composite materials belong to a group of materials that are difficult to analyze and have not been sufficiently researched so far in terms of their deformations as a result of various types of loads. A special case are fiber-reinforced materials, consisting of at least two materials. In these materials, it is particularly difficult to determine the actual range of elastic deformations, which is important for the design of structures. Based on previously conducted experimental research, it appears that it is possible to detect damage such as elastic or plastic cracks using the acoustic emission method [37,38]. Moreover, it allows you to monitor and identify damage, from the moment of its initiation, when the size of the cracks is still at the microscopic level, until visual changes such as fiber or matrix cracking [35,39,40]. This method can be used to determine the beginning of the deformation process of a composite material, which is of great importance, under fatigue loads and leading to broader and much more dangerous damage. The use of mechanical tests combined with the acoustic emission method allows not only to determine strength parameters, but also to detect delaminations and other degradation processes of composite materials. Matrix cracking, fiber cracking, and cracking at the interface between the matrix and the reinforcement can be identified, assessed, and analyzed using the parameters obtained when using this method [41,42,43,44]. The AE technique has unique advantages in examining the initiation and propagation of damage, as it allows for the description of damage and cracks in materials and the assessment of their resistance to these phenomena [45]. For more detailed damage monitoring, it is recommended to include an appropriate tool or signal in the analysis. In this research, the RMS parameter was used for analysis, based on the authors’ previous experience. Figure 2 shows a screenshot from the AE data software version R2023.1218.2 for an example sample.

**Figure 3:**
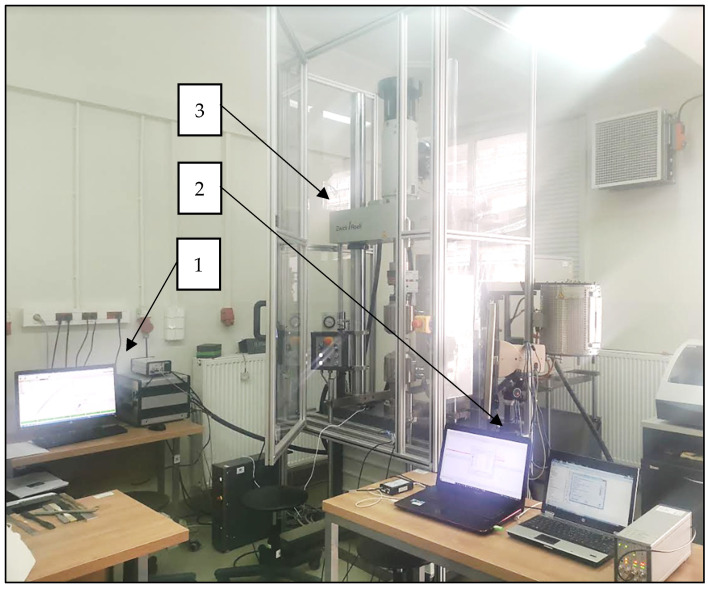
Measuring station: 1—computer with test expert II software Version 3.3, 2—computer with AE Win for USB software Version E5.30, 3—Zwick Roell testing machine.

**Figure 4:**
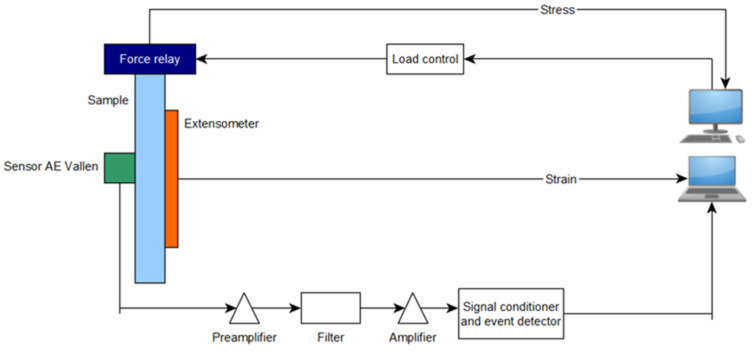
Diagram of measuring station [11,44].

**Figure 5:**
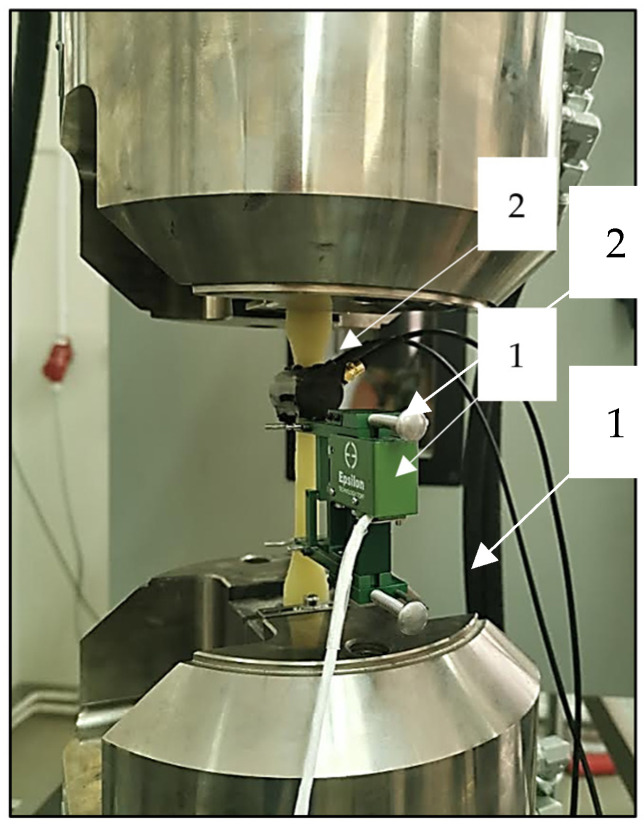
Sample undergoing testing: 1—extensometer, 2—AE sensor.

The tests were carried out using AE measuring instruments (Physical Acoustics Corporation, Princeton, NJ, USA) and a universal testing machine from Zwick Roel (Zwick Roell Group, Ulm, Germany). A piezoelectric sensor was installed on the tested specimens, which records the acoustic waves generated inside the material. These waves were converted by the sensor into an electrical signal, and then recorded in digital form by the recorder. The signal recorded was further processed and graphs of the effective value of the electrical signal (RMS), amplitude, and hits as a function of time were plotted [22]. The instruments consisted of a single channel recorder USB AE Node, type 1283 with bandpass 20 kHz–1 MHz, preamplifier with bandpass 75 kHz–1.1 MHz, AE-Sensor VS 150M (Vallen Systeme GmbH, Icking, (Munich), Germany), with a frequency range of 100–450 kHz, and a computer with AE Win for USB Version E5.30 software for recording and analyzing AE data (Physical Acoustics Corporation, Princeton, NJ, USA). Between the sensor and the surface of the specimen, a coupling fluid was used. The AE Sensor was fixed to the specimen by elastic tape. Figure 3, Figure 4 and Figure 5 shows the components of the measurement station [11,44].

Studies [46,47,48,49] have shown that the Kolmogorov-Sinai (K-S) metric entropy calculation method, performed on experimental data sets, is a useful tool in the statistical analysis of results, allowing the determination of additional changes occurring in materials. This method confirmed that the qualitative changes occurring at the construction threshold separating the elastic state from the plastic state correspond to a specific measurement point. In the testing machine–sample system, energy dissipation occurs during the test, and the deterministic chaos of data associated with this phenomenon causes entropy variability [44]. A fundamental and extremely important aspect in the use of this method is the preparation of input data and the accuracy of measurement tools. Local reductions in the metric entropy value obtained as a result of calculations performed on data sets (in this article, on strain sets) correspond to the points of significant qualitative changes occurring in the sample structure. It is assumed that local minima of the K-S metric entropy occur near the transition from the elastic to the plastic state in the tested material. The value of the stress, corresponding to the transition of the tested material from the elastic to the plastic state, is determined on the basis of the “critical” point. In testing metals, it was possible to determine the yield point using this method, while in testing composite materials, this method detects changes in structure that affect the strength parameters [44,49].

**Figure 6:**
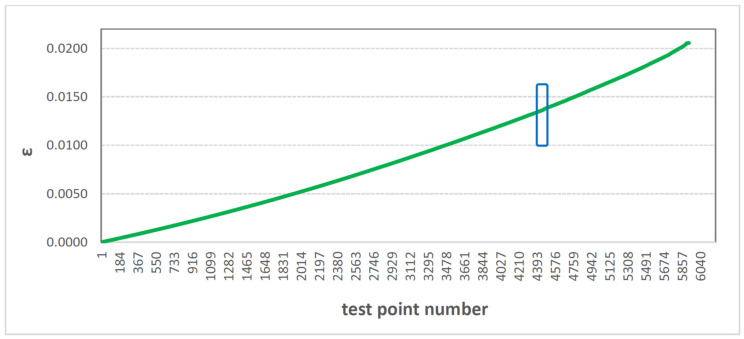
Graph of deformation depending on measurement points.

The sets of elongation values ε obtained during the static tensile test were subjected to calculations of the Kolmogorov-Sinai E_K-S_ metric entropy. To explain the E_K-S_ calculation method, we will use an example sample. During the static tensile test of the selected sample, 5929 measurement points were recorded by the machine with a sampling frequency of 50 Hz. Therefore, the test lasted 5929/50, i.e., less than 119 s. Figure 6 shows the set of elongation values ε as a function of subsequent measurement points (i.e., de facto, as a function of time).

Calculating the E_K-S_ entropy involves dividing the 5929-point set of ε values into intervals. In this case, the intervals contained 60 consecutive ε values, which corresponded to 60 consecutive elongation values recorded. The first interval of the set of values starts at the first measurement point and ends at the sixtieth measurement point. The next interval begins at the second measurement point and ends at the sixty-first.

Each 60-digit interval was then divided into 6 sub-intervals. The metric entropy value was calculated for each interval according to the principle defined by Kolmogorov-Sinai:

(1) $$E_{K-S}=-\sum_{i=1}^{N}p_{i}\ln p_{i}.$$

where:

p—probability,
i—state.

**Figure 7:**
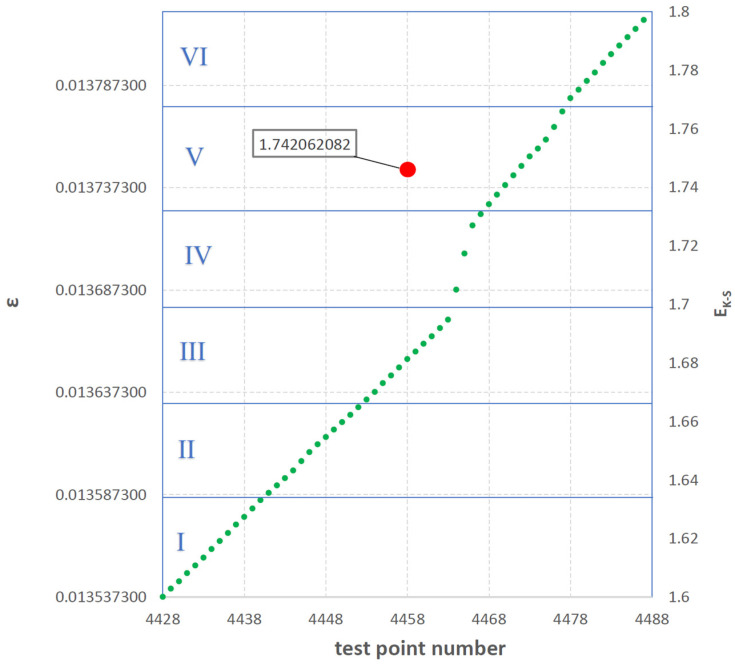
A fragment of the sample tensile curve containing one 60—digit interval of successively recorded elongation values ε and 6 marked sub-intervals of this interval.

Figure 6 shows one selected 60-digit range, which comes from the set of elongation values recorded during the tensile test of the selected sample. This interval is highlighted in Figure 6 with a blue rectangle. The selected interval was between the 4428th and 4488th measurement points. Figure 7 shows the elongation values ε included in the above-mentioned values in green compartment.

The lowest value of elongation ε recorded in this interval was 0.013537400 and the highest was 0.013823310. The interval was divided into six equal sub-intervals in terms of the value of absolute elongation (0.000047652 each), marked in the drawing with Roman numerals from I to VI and separated from each other by horizontal blue lines.

Table 2 presents a detailed method of calculating the E_K-S_ entropy for the selected interval shown in Figure 7. The probability p_i_ was calculated that an element of the adopted interval belongs to the appropriate subrange (from I to VI). This then made it possible to calculate the E_K-S_ entropy for the selected 60-digit interval according to Formula (1). The E_K-S_ entropy value of 1.742062082 was assigned to the midpoint of the calculated interval.

$$E_{K-S}=-\sum_{i=1}^{N}p_{i}\ln p_{i}=-\left(-0.331368961-0.321887582-0.311015703-0.180536680-0.298626578-0.298626578\right)=1.742062082$$

To calculate E_K-S_ for the entire set of elongation values ε recorded during the test, the above calculations should be performed on all intervals from the set of values. The number of recorded measurement points during the static tensile test for the selected sample was 5929 points. Metric entropy calculations were therefore performed for 5889 sets of 40-point intervals using Formula (1). The “Entropy K-S” software version 1.1 was used for calculations, which made it possible to calculate the metric entropy for each interval and obtain a set of metric entropy values. The metric entropy values for the adopted sample were presented graphically and compared with the stress diagram σ = f(ε) [46,47,48,49].

## 3. Results and Discussion

The strength parameters that were directly analyzed were tensile strength, Young’s modulus, and maximum deformation. Table 3 presents a summary of the results obtained from 30 samples (average), along with the standard deviation.

**Figure 8:**
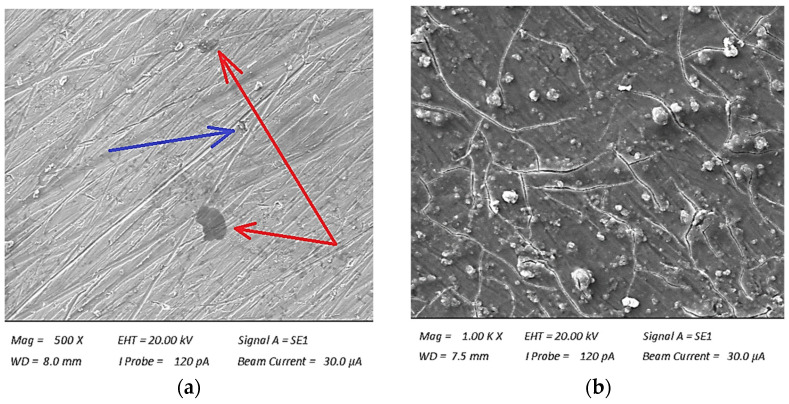
Observation of the surface using SEM for the sample: (**a**) A0R0, (**b**) A0R10.

Analyzing the obtained results presented in Table 3, the influence of the addition of recyclate on the decrease in the strength parameters of the composite material is noticeable. As a result of adding 10% of polyester–glass recyclate, decreases in tensile strength (UTS) by 32%, Young’s modulus by 16%, and deformation by 11% were observed. To verify the obtained parameters, the surfaces of the composites were observed using an EVO MA 15 scanning electron microscope (Carl Zeiss AG, Jena, Germany). Figure 8 shows structures using SEM to additionally illustrate the effect of recyclate on the tested composite material.

**Figure 9:**
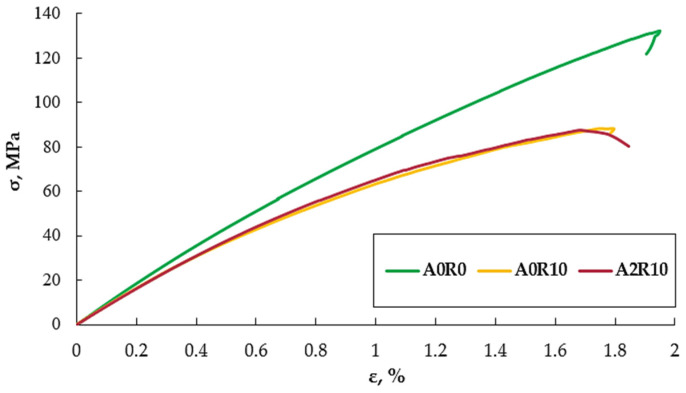
Examples of stress–strain curves for three tested materials.

Recyclate with granulation of ≤1.2 mm does not bind to the resin or dissolve in it, which causes delamination inside the composite and cracking of the matrix to occur much faster as a result of its addition. During the lamination process itself, the continuity of the fibers may be interrupted as a result of their damage by the recyclate added directly to the resin. An additional factor is the influence of the manufacturing method used. A very difficult aspect of manual lamination is the presence of air in the laminate and the difficulty in producing a laminate free of air bubbles (marked with a red arrow) and delamination on the surface (marked with a blue arrow). Adding polyester–glass recyclate certainly does not help, and even has a negative effect, thus increasing the size of air bubbles in the composite structure (Figure 8b). As a result of adding 2% of aluminum nanopowder to the composite with polyester–glass recyclate, a decrease in strength parameters was mainly observed. The tensile strength decreased by 7% compared to the material with the addition of recyclate only, and the deformation decreased by 5%. An increase in Young’s modulus by 5% was observed, which is a positive aspect of this composite material. The nanopowder did not significantly change the strength parameters when adding 2%. Much better strength parameters were expected because the nanopowder was very well combined with the resin, thus not having such an adverse effect on the composite as in the case of polyester–glass recyclate. The effect of aluminum nanopowder may be verified by using it in a smaller amount and in a much larger amount for comparative purposes. Figure 9 shows exemplary stress–strain curves for the three tested materials.

Analyzing the obtained exemplary tensile diagrams for the three tested materials, the influence of polyester–glass recyclate on the strength parameters and, unfortunately, on their decline, is noticeable. No noticeable improvement in parameters was observed due to the use of a 2% addition in the form of an aluminum nanofiller. The deformation increased slightly, but this was not the improvement in parameters that was expected.

**Figure 10:**
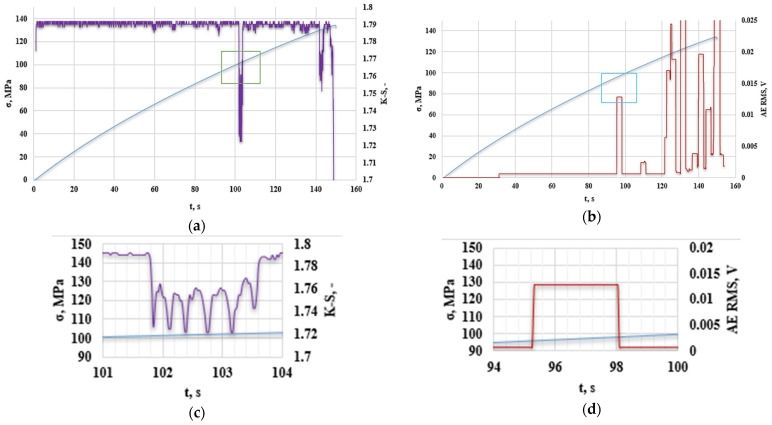
(**a**) K-S metric entropy as a function of time plotted on the stress–strain curves, (**b**) effective value of the electrical signal (RMS) as a function of time plotted on the stress–strain curve for an exemplary sample without the addition of recyclate (A0R0), (**c**) magnification of the first decrease in the entropy value, (**d**) magnification of the first increase in RMS [V].

Figure 10 presents an analysis of the obtained results of the K-S metric entropy and the acoustic emission method for an exemplary base sample without additives.

Based on previous research, it was observed that the decrease in K-S metric entropy indicates a qualitative change occurring in the structure of materials; the same applies to the increase in the RMS value from the acoustic emission method. The area of reduced entropy in the figure has been marked on the graph and enlarged. Stress–strain curves as a function of time are made due to the impossibility of comparing all analyzed parameters. Data obtained from the K-S metric entropy method and acoustic emission include time, thanks to which it was possible to correlate the obtained results. The graphs mark the first areas of reduced entropy (Figure 10a,c), the onset of which began in the 101st second of the test (up to approx. 104 s); in the case of acoustic emission, the first increase in RMS (Figure 10b,d) was observed at 94 s (up to approximately 98 s). These values do not differ significantly from each other and, interestingly, the acoustic emission method determines the change in the structure of the composite material slightly earlier than the metric entropy, which is because entropy calculations are performed on intervals of measurement points (in the case of the one analyzed in (Figure 10a,c), the sample interval was 9800 measurement points, where 50 measurement points correspond to 2 s and testing of the testing machine was set to 50 Hz). It was decided to verify the obtained results with the results of previous studies determining the type of damage at a given amplitude and frequency. Table 4 shows the characteristics of the emission signals of damage mechanisms in CFRP composites using a two-sensor approach and spectral clustering technique [33].

**Figure 11:**
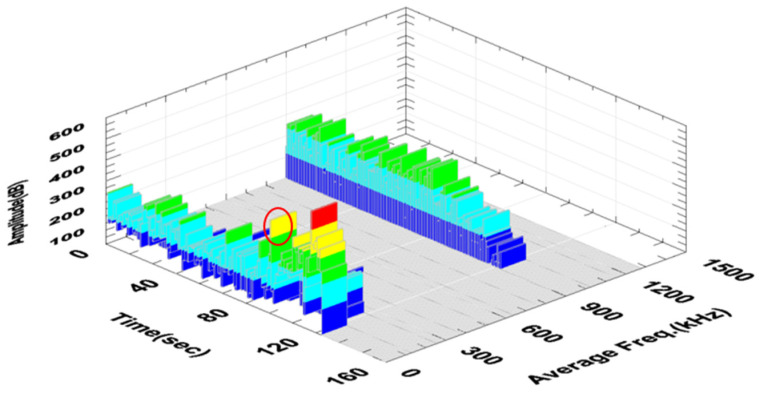
Change in the amplitude and frequency of the acoustic emission signal as a function of time for an exemplary sample made of a composite without additives (A0R0).

Figure 11 shows a graph of the change in the amplitude and frequency of the acoustic emission signal as a function of time for the analyzed sample (Figure 6) made of a composite without additives.

**Figure 12:**
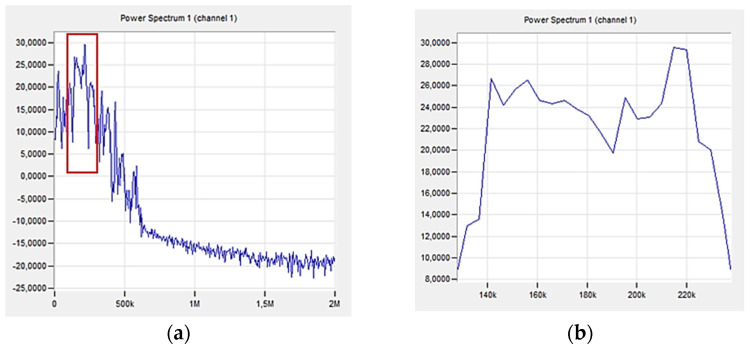
Change in acoustic emission signal power as a function of frequency for an exemplary sample made of a composite without additives (A0R0): (**a**) total spectrum, (**b**) enlargement of the selected area.

Figure 12 shows a graph directly from the acoustic emission software (PAC), which shows changes in the acoustic emission signal power as a function of frequency for an exemplary sample made of a composite without additives (A0R0).

The first significant increase in the amplitude value was observed in the 96th second of the test, as marked in Figure 10. The range of signal frequency changes was from 30 to 580 kHz, with the maximum power values recorded from 140 to 220 kHz. Based on the parameters obtained in previous tests (Table 4), the indicated strains correspond to the delamination and cracking of the matrix.

**Figure 13:**
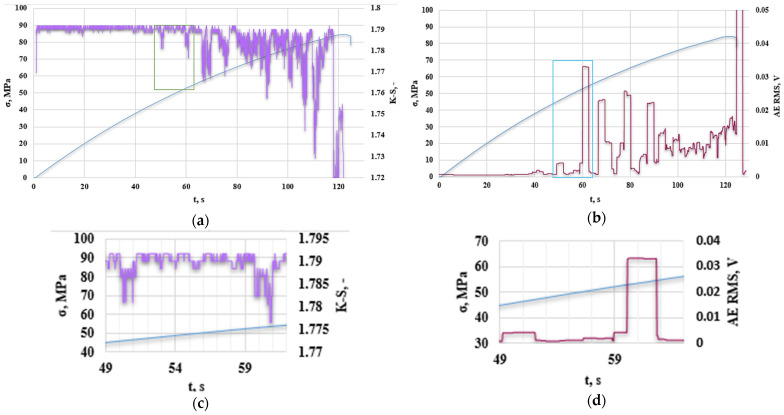
(**a**) K-S metric entropy as a function of time plotted on the stress–strain curve, (**b**) effective value of the electrical signal (RMS) as a function of time plotted on the stress–strain curve for an exemplary sample with 10% recyclate content (A0R10), (**c**) magnification of the first decrease in the entropy value, (**d**) magnification of the first increase of RMS [V].

Figure 13 shows the analysis using the K-S metric entropy results and the acoustic emission method for an exemplary sample with 10% addition of polyester–glass recyclate.

**Figure 14:**
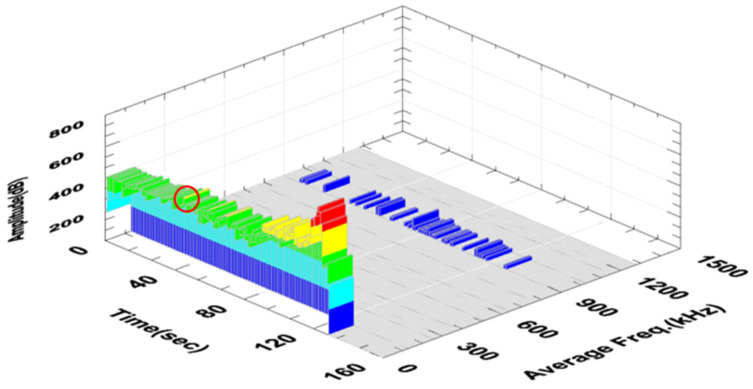
Change in the amplitude and frequency of the acoustic emission signal as a function of time for an exemplary sample made of a composite with 10% of recyclate (A0R10).

The graphs mark the first area of the K-S entropy drop (Figure 13a,c), which started in the 49th second of the test (up to approx. 60 s); in the case of acoustic emission, the first RMS increase (Figure 13b,d) was observed at 49 s (up to approximately 60 s). These values do not differ significantly from each other, but a completely different nature of the charts is noticeable, indicating that there are much more qualitative changes in the material. It can be concluded that the addition of recyclate has an impact on the nature of deformations, due to the process of combining it directly with the resin in the material during stretching, in addition to cracking of the matrix and cracking of the fibers, the recyclate moves inside the resin and cracks at the fiber or matrix. Additionally, due to the lack of appropriate adhesion between the resin combined with the recyclate and the reinforcement, there is no proper connection in this composite, which can also be determined by the decrease in strength parameters. To sum up, the nature of these changes in the material is different and occurs much faster, among others, to the cracking of the matrix, and the assessment of the destruction process is difficult due to the addition of recyclate, which is noticeable by the nature of the AE RMS (t) graph. Previous research allowed the determination of characteristic RMS values for destruction processes in the composite, such as matrix cracking or fiber cracking [41,42]. Figure 14 shows a graph of the change in the amplitude and frequency of the acoustic emission signal as a function of time for the analyzed composite sample (Figure 13) with 10% recyclate added.

**Figure 15:**
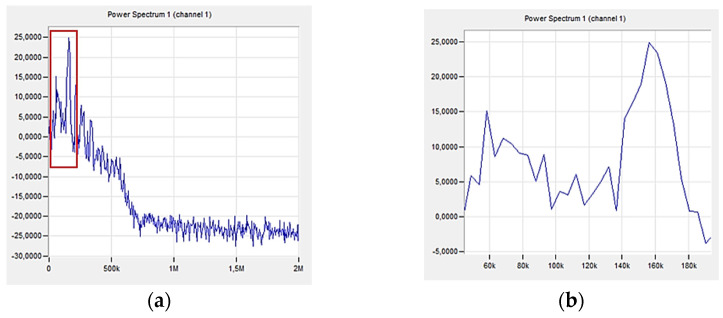
Change in acoustic emission signal power as a function of frequency for an exemplary sample made of a composite with 10% of recyclate (A0R10): (**a**) total spectrum, (**b**) enlargement of the marked area.

Figure 15 shows a graph of the change in acoustic emission signal power as a function of frequency for an exemplary sample made of a composite with 10% recyclate added.

The first significant increase in the amplitude value was observed in the 49th second of the test (marked in Figure 10. The range of signal frequency changes is from 40 to 340 kHz, with the maximum power values recorded from 60 to 160 kHz. Based on the results (Table 3), these frequencies indicate to delamination and cracking of the matrix. Lower frequencies of the maximum signal power indicate a less rapid destruction process compared to the sample without recyclate.

**Figure 16:**
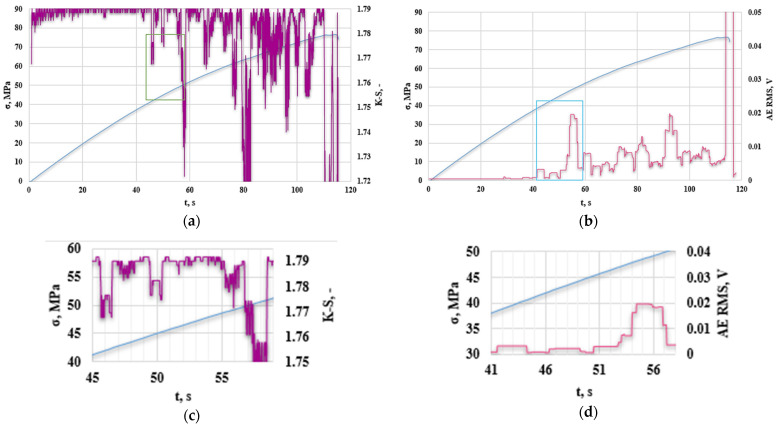
(**a**) K-S metric entropy as a function of time plotted on the stress–strain curve, (**b**) effective value of the electrical signal (RMS) as a function of time plotted on the stress–strain curve for an exemplary sample with 10% recyclate and 2% recyclate-nanopowder (A2R10), (**c**) magnification of the first decrease in the entropy value, (**d**) magnification of the first increase in RMS [V].

Figure 16 shows the analysis of the obtained results of the K-S metric entropy and the acoustic emission method for an example sample with 10% addition of polyester–glass recyclate and aluminum nanofiller.

**Figure 17:**
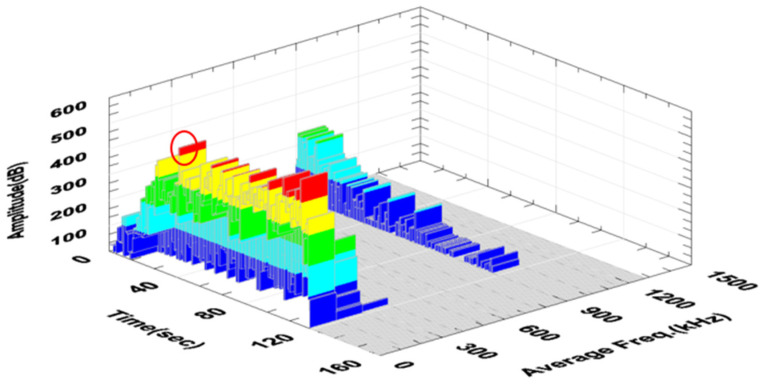
Change in the amplitude and frequency of the acoustic emission signal as a function of time for an exemplary sample made of a composite with 10% recyclate and 2% nanopowder (A2R10).

The graphs mark the first area of K-S entropy decrease (Figure 16a,c), which started in the 45th second of the test (up to approx. 60 s); in the case of acoustic emission, the first RMS increase (Figure 16b,d) was observed at 41 s (up to approximately 57 s). The behavior of this material under load differs from that of the material only with the addition of recyclate. In the case of the K-S metric entropy plot as a function of time, the obtained values are more or less similar to those only with the addition of recyclate. Based on the RMS graph, as a function of time, and compared to the material with the addition of recyclate, the RMS values are lower, the nature of the changes occurring in the material is different, and the amplitude values (averaged RMS) are lower. It can be concluded that despite the lack of increase in strength parameters, the addition of aluminum had a positive effect on the “homogeneity” of deformations and changes in the structure of the tested material. Figure 17 shows a graph of the change in the amplitude and frequency of the acoustic emission signal as a function of time for the analyzed composite sample (Figure 16) with 10% recyclate and 2% nanoadditive.

**Figure 18:**
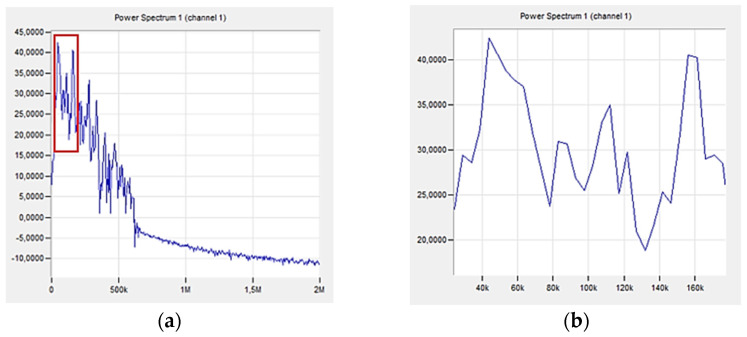
Change in acoustic emission signal power as a function of frequency for an exemplary composite sample with 10% recyclate and 2% nano-powder (A2R10): (**a**) total spectrum, (**b**) enlargement of the marked area.

Figure 18 shows a graph of the change in acoustic emission signal power as a function of frequency for an exemplary sample made of a composite with 10% recyclate and 2% nanoadditive.

The first significant increase in the amplitude value was observed in the 41st second of the test (in Figure 17, the first peak of the amplitude is marked in red). The range of signal frequency changes was from 40 to 600 kHz, with maximum power values recorded from 40 to 160 kHz. This indicates the deformation of the composite in the form of delamination and cracking of the matrix (Table 3). The maximum signal power value was recorded at frequencies in a similar range as for the sample with recyclate. This proves that the addition of aluminum powder has little impact on the nature of the composite destruction process compared to the sample with recyclate but without aluminum. For all samples considered, the recorded high-frequency signals were not significant and were treated as noise.

**Figure 19:**
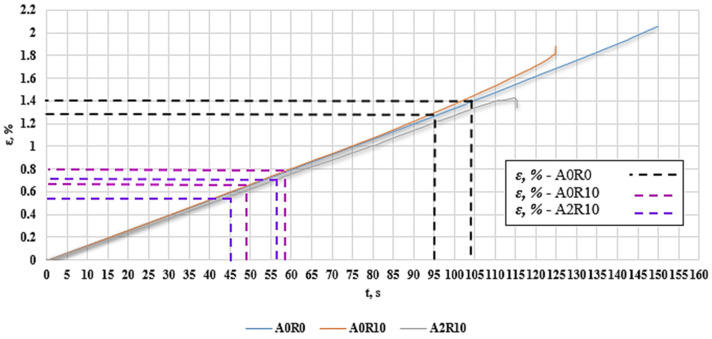
Plot of deformation as a function of time ε (t), with plotted values obtained by applying K-S metric entropy and acoustic emission (RMS).

Figure 19 shows the strain versus time ε (t) plots, with the values obtained by applying the K-S metric entropy and acoustic emission (RMS).

**Figure 20:**
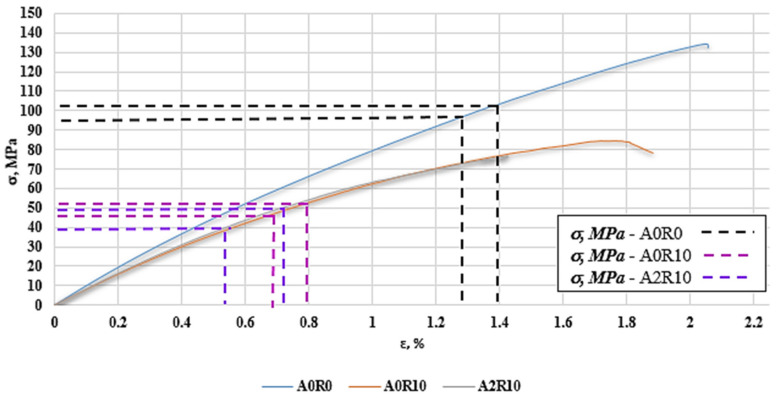
Stress versus strain σ (ε) with plotted values obtained by applying K-S metric entropy and acoustic emission (RMS).

The time values for the first decreases in the K-S entropy and the increase in the RMS value from the graphs (Figure 10, Figure 13 and Figure 16) are plotted on a strain plot as a function of time in order to determine the strain values for the changes taking place. In the case of samples without the addition of recyclate—A0R0—the deformation was approximately 1.3–1.4%. For a sample with 10% recyclate content—A0R10—the deformation was approximately 0.7–0.8%. For a sample with 10% recyclate and 2% aluminum nanopowder—A2R10—the deformation was approximately 0.55–0.75%. These values were plotted on a graph of stresses as a function of strains (Figure 20) in order to determine the values of strength parameters that can be used in composites as permissible stresses for the design of structures.

On the basis of the plotted strain values, the stress values corresponding to the first changes occurring in the structure of the tested materials during the static tensile test were read from the graph. For a sample without additives, the stress values were 95–103 MPa, which is approximately 70% of UTS for this sample. In the case of a sample with 10% recyclate content, the obtained stresses were 47–52 MPa, which was approximately 55% of UTS. However, for a sample with 10% recyclate and 2% nanopowder content, the obtained stress values were 40–50 MPa, which was approximately 60% of UTS. Table 5 presents statistics of the results obtained for the five tested samples from each material, averaging the lowest and highest strain values (strain value range) and the lowest and highest stress values (stress value range). The %UTS was also averaged based on the five tested samples, defined as a utility parameter (composite allowable stress).

## 4. Conclusions

As a result of adding 10% of polyester–glass recyclate, a decrease in strength parameters was observed in the static tensile test. Recyclate with a granulation of ≤1.2 mm does not bind to the resin or dissolve in it, which causes deformation in the composite to occur much faster as a result of its addition. Adding an additional 2% of aluminum nanopowder to the composite with polyester–glass recyclate resulted in a slight decrease in strength parameters. An increase in Young’s modulus by 5% was observed. The nanopowder did not significantly change the strength parameters when adding 2%. The destruction process was analyzed on the tested materials, with emphasis on its initial phase, using the acoustic emission method and K-S metric entropy. The obtained data made it possible to determine not only the stress values for the first changes in the structure of composite materials, but also their nature by analyzing the frequencies for these points. This allows us to conclude that the acoustic emission method is extremely useful in this analysis and repeatable when using the same type of reinforcement. The stress values read from the chart based on the analysis performed allow determining the safety ranges for these composite materials. For a sample without additives, the stress values range from 95–103 MPa, which is approximately 70% of UTS for this sample. In the case of a sample with 10% recyclate content, the obtained stresses are 47–52 MPa, which is approximately 55% of UTS. However, for a sample with 10% recyclate content and 2% nanopowder content, the obtained stress values range from 40–50 MPa, which is approximately 60% UTS. There was no increase in the strength parameters of the composite with recyclate as a result of the addition of aluminum nanofiller; however, its addition influenced the operational parameters, which is reflected in a 5% increase in the UTS value (from 55% to 60%). This may have a positive impact on the parameters used during the modeling process. These values can be extremely useful when designing structures, after their thorough verification using fatigue tests, which will be the next step in the research.

## Figures and Tables

**Table 1:** List of components of tested composites.

Sample	Resin	Glass Fiber	Gamma-Phase Aluminum Nanopowder	Polyester–Glass Recyclate
	%	%	%	%
A0R0	60	40	0	0
A0R10	60	30	0	10
A2R10	60	28	2	10

**Table 2:** A detailed method of calculating the E_K-S_ entropy for the selected 60-digit range.

	Six Adopted Sub-Ranges of the Selected Range					
	I	II	III	IV	V	VI
	min sub-range 0.013537400	min sub-range 0.013585052	min sub-range 0.013632703	min sub-range 0.013680355	Min sub-range 0.013728007	Min sub-range 0.013775658
Adopted 60—digit interval from the set of extensions ε	0.013537400 0.013541397 0.013544890 0.013548909 0.013552707 0.013556491 0.013560667 0.013564632 0.013568523 0.013572643 0.013576444 0.013580532 0.013584602	0.013588142 0.013591788 0.013595396 0.013599111 0.013603684 0.013608099 0.013611917 0.013615487 0.013619061 0.013622743 0.013626323 0.013630034	0.013633789 0.013637494 0.013641752 0.013645518 0.013649446 0.013653519 0.013657227 0.013661062 0.013664697 0.013668638 0.013672831	0.013687458 0.013705087 0.013718830 0.013724324	0.013729168 0.013733916 0.013738563 0.013743314 0.013747923 0.013752592 0.013756413 0.013760804 0.013766979 0.013774511	0.013781058 0.013785108 0.013789486 0.013793529 0.013798223 0.013802508 0.013806735 0.013810797 0.013814831 0.013819282
	max sub-range < 0.013585052	max sub-range < 0.013632703	max sub-range < 0.013680355	max sub-range < 0.013728007	max sub-range < 0.013775658	max sub-range < 0.013823310
*p_i_*	0.21666666	0.2	0.18333333	0.06666666	0.16666666	0.16666666
ln *p_i_*	−1.529395205	−1.609437912	−1.696449289	−2.708050201	−1.791759469	−1.791759469
*p_i_* ln *p_i_*	−0.331368961	−0.321887582	−0.311015703	−0.180536680	−0.298626578	−0.298626578
E_K-S_	1.742062082					

**Table 3:** Summary of the results obtained from 30 samples (average).

Sample	UTS	E	ε
	Mpa	Mpa	%
**A0R0**	128.3	9169	1.87
**Standard deviation**	8.4	439	0.12
**A0R10**	87.0	7691	1.67
**Standard deviation**	5.7	369	0.12
**A2R10**	80.5	8057	1.59
**Standard deviation**	6.6	575.3	0.18

**Table 4:** Characterization of reported failure mechanisms [33].

Failure Mechanism	Amplitude	Peak Frequency
	dB	kHz
Delamination/Matrix cracking	40–94	50–200
Fiber/matrix debonding	40–70	200–400
Fiber failure	>80	400–600
Fiber pull-out	>60	>700

**Table 5:** Summary of statistics for the results obtained based on the analysis of five samples of the tested composites.

Sample	Strain Value Range	Stress Value Range	UTS
	%	Mpa	%
A0R0	1.27–1.45	93–110	72
A0R10	0.68–0.89	49–54	56
A2R10	0.52–0.72	42–49	61

## Data Availability

The data presented in this study are available on request from the corresponding author. The data are not publicly available due to large quantities.
